# Phylogenetic analysis of porcine circovirus 2 and its related viruses

**DOI:** 10.3389/fvets.2025.1711361

**Published:** 2025-12-08

**Authors:** Libin Wen, Han Wen, Qi Xiao, Jianping Xie, Kongwang He

**Affiliations:** 1 Institute of Veterinary Medicine, Jiangsu Academy of Agricultural Sciences, Nanjing, China; 2 Key Laboratory of Veterinary Biological Engineering and Technology, Ministry of Agriculture and Rural Affairs, Nanjing, China; 3 Jiangsu Co-Innovation Center for Prevention and Control of Important Animal Infections Diseases and Zoonoses, Yangzhou University, Yangzhou, China; 4 Jiangsu Key Laboratory for Food Quality and Safety—State Key Laboratory Cultivation Base of Ministry of Science and Technology, Nanjing, China; 5 GuoTai (Taizhou) Center of Technology Innovation for Veterinary Biologicals, Taizhou, China; 6 UltraRISC Technology Co., Ltd., Shanghai, China

**Keywords:** PCV2, porcine circovirus-like virus, porcine circovirus-like mini agent, evolutionary, ancestral sequence

## Abstract

**Introduction:**

Porcine circovirus (PCV) belongs to the family *Circoviridae*. Four conventional PCVs (PCV1 to PCV4) have been discovered over time. PCV1 and PCV2 exhibit higher nucleotide identity compared to PCV3 and PCV4. PCV2, in particular, has caused porcine circovirus-associated diseases (PCVAD), posing a significant threat to the global swine industry. Subsequently, unconventional PCVs, including porcine circovirus-like viruses and porcine circovirus-like mini agents, with genome sequences highly homologous to the PCV2 capsid protein sequence, have been reported.

**Methods:**

This advancement facilitates phylogenetic analysis of these sequences to elucidate the evolutionary history of PCV2, although its true origin remains undetermined.

**Results and discussion:**

Based on the findings, it is speculated that PCV2 may have originated from ancestral non-coding DNA sequences. This speculation provides new insights into the evolutionary question of which nucleic acid and protein first emerged.

## 1 Introduction

Porcine circoviruses (PCVs) are members of the genus Circovirus within the Circoviridae family, characterized by a covalently closed circular, single-stranded genomic DNA ranging from approximately 1.7 to 2.0 kb in length. They possess icosahedral capsid proteins with a diameter of approximately 17 nanometers, making them the smallest autonomously replicating viruses. To date, four conventional types have been identified: PCV1, PCV2, PCV3, and PCV4. PCV1, first identified in the early 1970s, is considered nonpathogenic (1–3). Conversely, PCV2, PCV3, and PCV4 are recognized as being associated with several clinical disease syndromes in pigs, collectively referred to as porcine circovirus disease or porcine circovirus-associated disease (4–9). Of these, PCVD associated with PCV2 has caused significant economic losses to the global pig farming industry, prompting more comprehensive and systematic research on PCV2.

PCV2 was first identified in Canada in the early 1990s, and later retrospectively detected in samples collected in 1962 (10). The PCV2 genome contains approximately 1,770 nucleotides and includes a palindrome stem-loop structure, with a conserved nonanucleotide sequence (CAGTATTAC) located within the intergenic region between two major open reading frames (ORFs) oriented in opposite directions, similar to other PCVs. ORF1 encodes the replicase protein (Rep and Rep’) (11), whereas ORF2 encodes the capsid protein (Cap) (12). Cap is the sole structural protein of PCV2, playing a crucial role in clathrin-mediated endocytosis, actin- and small GTPase-dependent pathways for viral entry into host cells, and immune evasion (13, 14). PCV2 shares a high nucleotide identity with PCV1 but exhibits low nucleotide identity with PCV3 and PCV4. Based on complete genomic analysis, PCV2 shares less than 80% genomic identity with PCV1, 46.8% with PCV3, and 51.5% with PCV4 (7, 15, 16).

The high genetic diversity of PCV2 was recognized following its discovery, as it exhibits the highest evolutionary rate among DNA viruses (17). Currently, at least nine distinct genotypes (a through i) have been reported, based on three criteria: a maximum within-genotype p-distance of 13%, minimum cluster internal node bootstrap support higher than 70%, and at least 15 available sequences (18, 19).

To date, PCV2 has undergone two major genotype shifts: the first from PCV2a to PCV2b, and the second from PCV2b to PCV2d (20–22).

A distinct PCV species, designated as porcine circovirus-like viruses (P1, P2, and P4 viruses), has been identified in China in pigs with various clinical signs. These viral genomes are less than 1,000 nucleotides in length, with the smallest porcine circovirus-like virus P1, containing only 648 or 649 nucleotides. The P1 virus has many natural hosts, including pigs, rabbits, goats, cattle, yaks, dogs, and cats. In contrast to the conventional PCVs mentioned above, these viruses do not contain sequences similar to Rep but only sequences highly homologous to the Cap of PCV2. PCV2 Cap consists of 233, 234, or 238 amino acids. The P1 virus is divided into two genotypes based on genome length. The P1 Cap with 648 nt consists of 114, or122 amino acids, while the P1 Cap with 649 nt consists of 163 amino acids. The P2 Cap is composed of 233 amino acids, and the P4 Cap is composed of 167 amino acids. Those viral Cap sequences with 163, 167, or 233 aa are highly homologous to the PCV2 capsid protein sequence. Although the N-terminal region of the P1 Cap with 114 or 122 aa is similar to a corresponding region in the PCV2 Cap, the C-terminal region shows low homology with the PCV2 Cap sequence due to a single base deletion.

In 2019, porcine circovirus-like mini agents were detected in pigs and cows in China. To date, four types of porcine circovirus-like mini agents have been discovered: PCVL258, PCVL264, PCVL201, and PCVL347. PCVL258 and PCVL264 have been detected in pigs, cats, and/or dogs, whereas PCVL201 and PCVL347 have been detected exclusively in cattle. Although their genome sequences are highly homologous to those of PCV2 and porcine circovirus-like viruses, they are shorter and do not encode any proteins. Research has indicated that some of these mini agents may be involved in metabolic pathways and are associated with human diseases, such as degenerative neurological disorders (23–25).

Gibbs and Weiller speculated that PCVs originate from the recombination of a plant virus and a vertebrate-infecting virus, based on Rep sequence analysis (26). Some scholars suggest that PCV1 and PCV2 evolved from avian circovirus (17). Recent reports indicate that PCV1 and PCV2 are closely related to bat-clade 2 circoviruses, PCV3 is closely related to bat-clade 1 circoviruses, and PCV4 is closely related to mink circoviruses (7, 27). However, the true origin of PCVs remains unclear. Currently, porcine circovirus-like viruses and porcine circovirus-like mini agents have been detected exclusively in China, and their distribution in other countries remains unknown. Nevertheless, the emergence of these viruses and agents provides valuable materials for studying the origin of PCV2.

## 2 Materials and methods

### 2.1 Alignment and phylogenetic analysis

The 50 available full genome sequences of PCV1, PCV2, PCV4, porcine circovirus-like viruses, and porcine circovirus-like mini agents were downloaded from GenBank and used as reference sequences (Table 1). The genomic sequences of circoviruses selected for this study were based on the following three criteria: obtained from published literature; or a maximum intragenotype p-distance value of 0.02; and originating from as many different countries as possible. Each sequence was annotated with the accession number, sampling country, host species, and collection date. Complete Cap sequences of PCV1, PCV2, and porcine circovirus-like viruses were also compiled.

**Table 1:** Information on the PCV strains used in this study.

virus	Codename	Accession number	Strain name	Sampling country	Host specie	Collection date	Reference
P1	01	EF514716	HB1	China	Porcine	2005	(24)
P1	02	JN040278	CZJT	China	Porcine	2011	(24)
P1	03	KU243695	JSPZ	China	Porcine	2015	(24)
P1	04	KU323639	SYD1207	China	Porcine	2015	(24)
P1	05	KU356938	E061362	China	Porcine	2015	(24)
P1	06	OL581721	JS02c	China	Porcine	2014	(24)
P2	07	EF514717	HB2	China	Porcine	2005	(44)
P4	08	MF802834	SQ01	China	Porcine	2017	(45)
PCV1	09	MN508363	LV34	Brazil	Porcine	2016	(46)
PCV2a	10	AF381175	BF	China	Porcine	2001	(45)
PCV2a	11	KC618389	STRUVE	USA	Porcine	2008	(47)
PCV2a	12	AB072303	No.35	Japan	Porcine	2009	-
PCV2a	13	KX828218	KU-1615	South Korea	Porcine	2016	-
PCV2b	14	OR533482	JS05	China	Porcine	2014	(48)
PCV2b	15	KY388471	DE38316-13	Germany	Porcine	2013	(49)
PCV2b	16	HQ591368	126–07-5	Croatia	Porcine	2007	-
PCV2b	17	KP420201	ZrLd_wb UKR	Poland	Porcine	2012	-
PCV2c	18	EU148503	DK1980PMWSfree	Denmark	Porcine	1980	(18, 30)
PCV2c	19	KJ094599	PM163	Brazil	Porcine	2010	(50)
PCV2c	20	EU148504	DK1987PMWSfree	Denmark	Porcine	1987	(18, 30)
PCV2c	21	EU148505	DK1990PMWSfree	Denmark	Porcine	1990	(30)
PCV2d	22	OR533478	JS26	China	Porcine	2014	(48)
PCV2d	23	MK504418	KSU-IA-2018-PCV2-45	USA	Porcine	2018	-
PCV2d	24	KT336599	Hau7	Viet Nam	Porcine	2014	-
PCV2d	25	PQ468872	12,017	Italy	Porcine	2023	-
PCV2e	26	KT795290	USA/45358/2015	USA	Porcine	2015	(31)
PCV2e	27	MF589524	PCV2-CN/FuJian-625-2017	China	Porcine	2017	(51)
PCV2e	28	MT188579	PCV2e/KOR/198326/2019	South Korea	Porcine	2019	-
PCV2e	29	KT795287	MEX/41238/2014	Mexico	Porcine	2014	(31)
PCV2f	30	MF278779	GD18/1999	China	Porcine	1999	(52)
PCV2f	31	LC004750	MZ-5	India	Porcine	2013	(18)
PCV2f	32	KT369067	Papuan 04.1	Indonesia	Porcine	2013	-
PCV2f	33	HQ591379	1,313-09-10	Croatia	Porcine	2009	-
PCV2g	34	JX099786	P2425NT	Viet Nam	Porcine	2008	(53)
PCV2g	35	HQ395060	10BJ-2	China	Porcine	2012	(54)
PCV2g	36	KP420197	ZrBd_wb UKR	Ukraine	Porcine	2010	(18)
PCV2g	37	AY713470	-	Germany	Porcine	2005	(55)
PCV2h	38	MH465473	GXYL1208	China	Porcine	2012	-
PCV2h	39	JQ181592	BG0-1	Viet Nam	Porcine	2011	(18, 53)
PCV2h	40	KJ729074	PCV2Izn-218-13	India	Porcine	2013	(56)
PCV2h	41	EU302141	INDON07-P09-07-07Lg2	Indonesia	Porcine	2008	(57)
PCV2i	42	OR533476	JS48	China	Porcine	2014	(48)
PCV2i	43	KM116515	Buffalo3	China	Beef	2013	(58)
PCV2i	44	MK347370	HLJ290315	China	Porcine	2015	-
PCV2i	45	GU370063	XJ0901	China	Porcine	2009	(59)
PCVL201	46	MH257309	JS01	China	Cattle	2016	(60)
PCVL258	47	KY270811	PCVL258	China	Porcine	2014	(60)
PCVL264	48	KY270812	PCVL264	China	Porcine	2014	(60)
PCVL347	49	MH257310	JS02	China	Cattle	2017	(60)
PCV4	50	MT311854	PCV4/GX2020/FCG49	China	Porcine	2018	(61)

The full genome sequences were aligned using the ClustalW method implemented in MEGA 7 (28).

The PCV1 genome has a nucleotide length of 1,759, the PCV4 genome has a nucleotide length of 1770, while the PCV2 genome ranges from 1,767 to 1,777 nucleotides. The porcine circovirus-like virus genome measures between 648 and 993 nucleotides in length, and the porcine circovirus-like mini-agent genome contains 201 to 347 nucleotides. Due to significant differences in nucleotide sequence lengths among these PCV strains, the aligned sequences were divided into four categories for processing: the first category comprises the complete genome sequence alignment without trimming; the second category is based on the alignment of sequences matching the length of the P1 virus genome (649 nt); the third category aligns sequences based on the length of the PCVL347 genome (347 nt); and the fourth category includes sequences from which non-homologous regions at both ends were removed.

The aligned sequences, whether trimmed or untrimmed, were then used for phylogenetic analysis. Phylogenetic trees were constructed and tested using the Maximum Likelihood (ML), Neighbor-Joining (NJ), and Minimum-Evolution (ME) methods in MEGA 7, with a bootstrap of 1,000 replicates.

### 2.2 Divergence time estimation

Based on the available ML, NJ, and ME phylogenetic trees, the evolutionary relationship of taxa (timetree) was constructed for the estimation of divergence times using the RelTime method in MEGA 7 (29). To achieve this, we designated PCV1 or PCV4 as the outgroup and assigned 14 years to calibrate the divergence between PCV2a and PCV2d as the first calibration point; and 34 years to calibrate the divergence between PCV2c and PCV2e as the second calibration point, both derived from the fossil record (10, 22, 30, 31).

Meanwhile, MCMCTree in BEAST 1.10.4 (32) was also employed for the Bayesian serial coalescent approach to estimate divergence times for 46 candidate PCV nucleotide sequences. For this purpose, the best substitution model was selected based on Hasegawa-Kishino-Yano (HKY); the molecular clock was selected as an uncorrelated relaxed clock; the tree priors used were either the Bayesian SkyGrid model or the Bayesian Skyline model. The divergence time of candidate PCV nucleotide sequences was estimated using substitution rates previously estimated for PCV (17, 24, 33). All parameters were estimated by performing a Markov chain Monte Carlo (MCMC) sampling over 10 million generations, with parameters sampled every 10 thousand generations. Mixing and convergence of the runs were assessed using Tracer 1.7^1^ after a 10% burn-in, with results accepted only if the estimated sample size was greater than 200. Parameters were summarized in terms of mean and 95% Highest Posterior Density (HPD) after excluding a burn-in equal to 10% of the run length. A maximum clade credibility (MCC) tree was also reconstructed using the treeAnnotator tool from the BEAST package. FigTree (version 1.4.4) was used to visualize the MCC tree.

### 2.3 Reconstructions of ancestral sequences

Based on the phylogenetic tree, the Ancestors function in MEGA 7 was used to reconstruct the most probable ancestral sequence of PCVs using the Maximum Likelihood method and the Tamura-Nei model. Regions containing gaps and missing data were eliminated. For each branch, nucleotides with more than 50% coverage across the species were considered reliable. We predicted the secondary structure of the ancestral sequence using three relevant websites: (1) http://www.unafold.org/mfold, (2) https://www.vectorbuilder.cn/tool/dna-secondary-structure.html, and (3) https://rna.tbi.univie.ac.at/cgi-bin/RNAWebSuite/RNAfold.cgi.

### 2.4 Three-dimensional (3D) structures modeling

Three-dimensional (3D) structures of the Cap proteins of P1 (GenBank No. EF514716; OL581721), P2 (GenBank no. EF514717), and PCV2d (GenBank no. OR533478) were predicted using the I-TASSER online tool^2^ (34).

The viral surfaces and structures were visualized using PyMOL (v3.0.3) (The PyMOL Molecular Graphics System, Version 3.0 Schrödinger, LLC). Mutations in the Cap proteins were compared and analyzed.

## 3 Results

### 3.1 Evolutionary analysis

**Figure 1:**
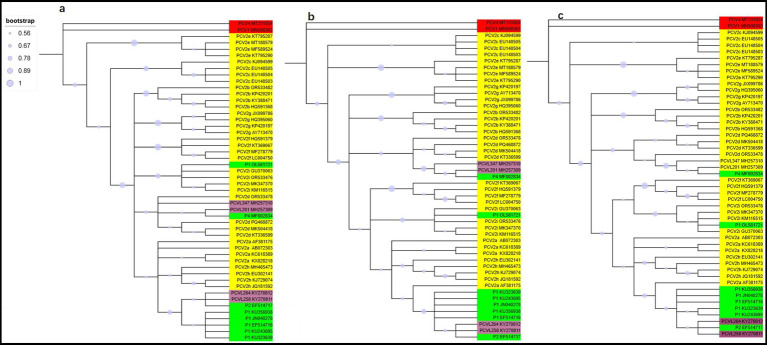
Phylogenetic trees of circoviruses based on 50 trimmed genomic sequences with redundant sequences removed from both ends. Trees were reconstructed using maximum likelihood **(a)**, neighbor-joining **(b)**, and minimum-evolution **(c)** methods in MEGA 7.0, employing the Tamura-Nei, p-distance, and p-distance models, respectively, with 1,000 bootstrap replicates and bootstrap values greater than 50%. Virus strains are followed by GenBank accession numbers. PCV1 or PCV4 is marked in red, PCV2 is marked in yellow, porcine circovirus-like viruses (P1, P2, and P4) is marked in green, and porcine circovirus-like mini agents (PCVL258, PCVL264, PCVL201, and PCVL347) is marked in purple.

To gain a clearer understanding of the evolutionary origin of PCV2, we analyzed 36 PCV2 strains representing different genotypes, along with other circoviruses, including one PCV1 strain, one PCV4 strain, six porcine circovirus-like virus P1 strains, one P2 strain, one P4 strain, and porcine circovirus-like mini agents (PCVL201, PCVL258, PCVL264, and PCVL347). The mean pairwise genetic distance across the full genome was 0.178% (interval: 0.00–1.13%) for the full genome. To trace the origin of PCV2, we reconstructed a phylogenetic tree based on both trimmed and untrimmed sequences, using the ML, NJ, and ME methods. The results indicated that the phylogenetic trees constructed using ML, NJ, and ME methods, whether based on trimmed or untrimmed sequences, exhibited similar topologies. PCV1 or PCV4 formed a distinct branch separate from other PCVs. PCV2 was classified into nine genotypes: PCV2a is closely related to PCVL258, PCVL264, P2, and P1 strains with capsid protein composed of 114, or 122 amino acids; PCV2i is closely related to P1 strain with capsid protein composed of 163 amino acids, PCV2d is closely related to PCVL201, PCVL347, and P4 strains. The evolutionary relationship between PCV2c and PCV2e is slightly more distant from other PCV2 genotypes (Figure 1; Supplementary Figure 1).

### 3.2 Timetree of PCV clusters

**Figure 2:**
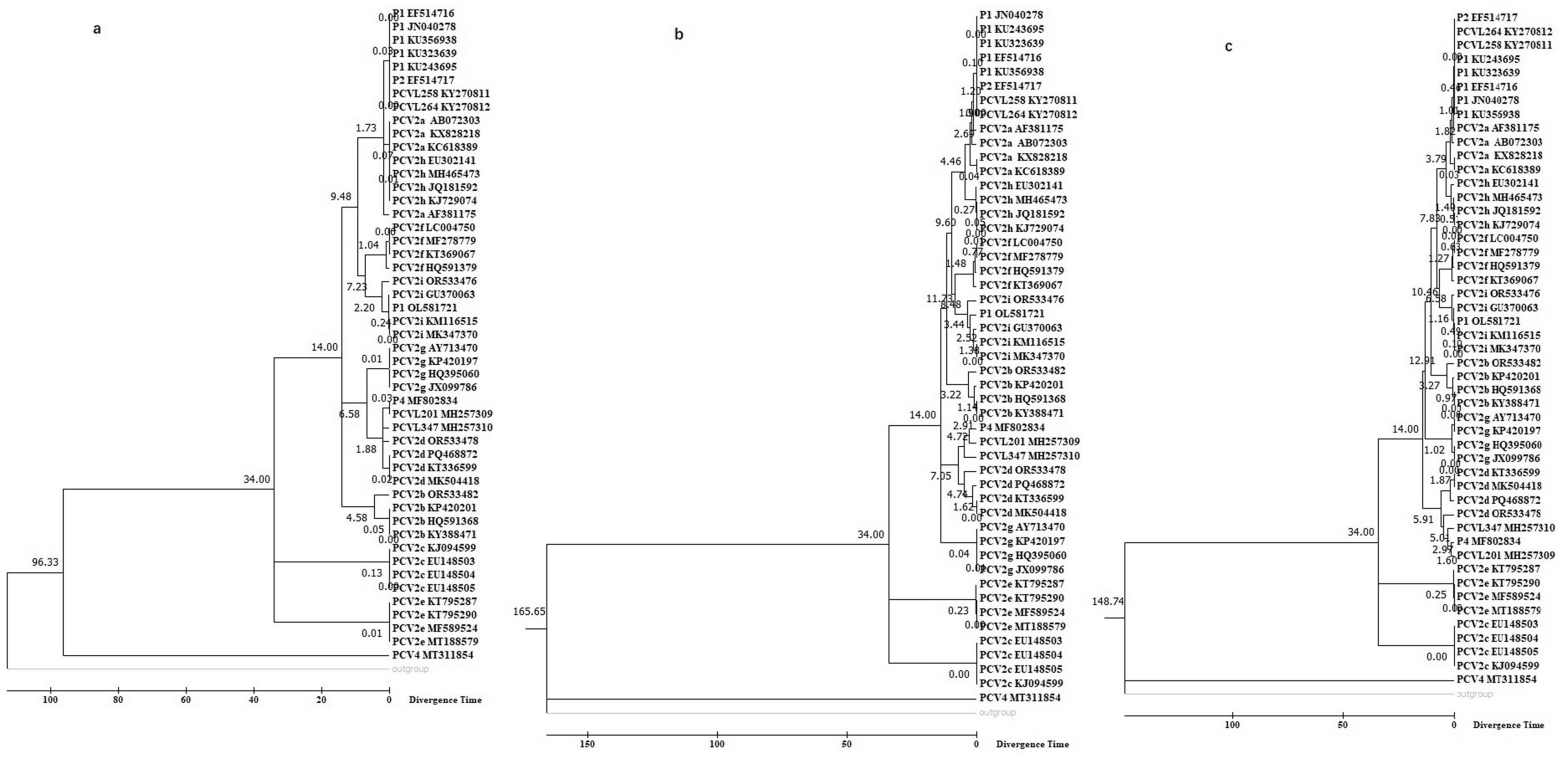
Molecular timetree of PCV clusters constructed using the RelTime method in MEGA 7.0. The analysis was based on 50 trimmed genomic sequences with redundant sequences removed from both ends, employing maximum likelihood **(a)**, neighbor-joining **(b)**, and minimum-evolution **(c)** methods. The PCV1 sequence was used as the outgroup, and two reference dates (14 and 34 years) were used for calibration.

Based on varying sequence lengths and statistical methods, we present three timetrees from the phylogeny in Figure 2; along with nine additional timetrees in Supplementary Figure 2.

We utilized fossil records from 1985 for PCV2a and 1999 for PCV2d as the first calibration point; and from 2014 for PCV2e and 1980 for PCV2c as the second calibration point. Divergence time analysis with these calibrations indicates that PCV2a, PCVL258, PCVL264, P2 strain, and P1 strains with 114 or 122aa Cap emerged simultaneously, then followed by PCV2a; PCV2d, PCVL201, and PCVL347 appeared at a similar time; and PCV2i and P1 strain with 163aa Cap emerged around the same time. Both PCV2g and PCV2d are younger than PCV2a, PCV2i, PCV2b, PCV2f, and PCV2h, while PCV2c and especially PCV2e evolved more recently compared to other genotypes of PCV. Whether PCV1 or PCV4 is used as the outgroup, the PCV timetree generated by phylogenetic analysis using MEGA software is consistent.

Differences in viral divergence times are observed depending on whether the Bayesian SkyGrid model or Bayesian Skyline model is used. Regardless of which model is used for the divergence analysis, three main groups can emerge: Group A includes all P1 strains, P2 strain, PCV2a, PCV2f, PCV2h, and PCV2i; Group B includes PCV2d, PCV2g, PCV2b, and P4 strain; Group C includes PCV1, PCV2c, PCV2e, and PCV4.

**Figure 3:**
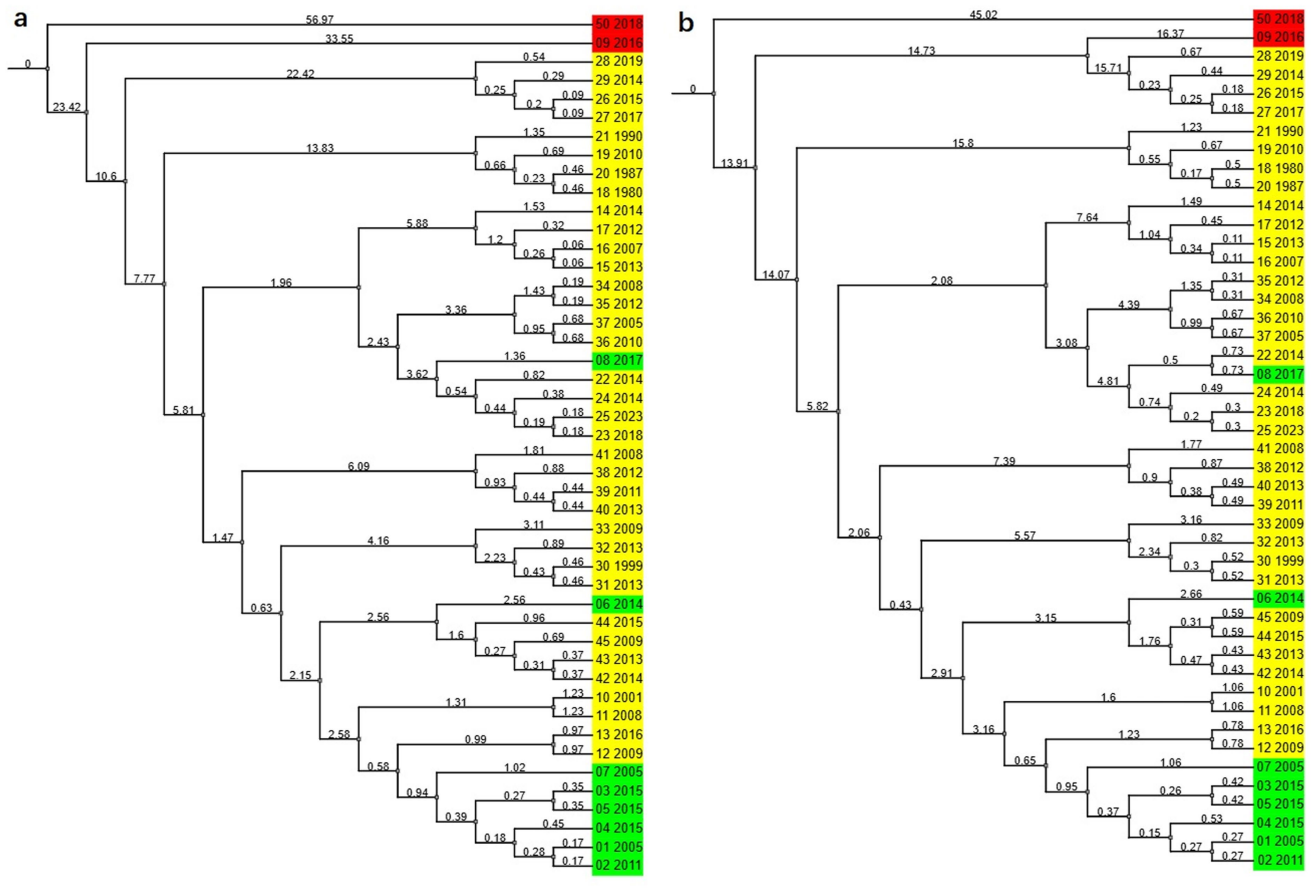
Molecular evolutionary history of PCVs calibrated with two fossil priors in BEAST. MCC trees were constructed using the Bayesian SkyGrid model **(a)** or the Bayesian skyline model **(b)**, with an uncorrelated relaxed clock applied to the trimmed nucleotide sequences of 46 PCVs. The color red represents PCV1 or PCV4, yellow represents PCV2, and green represents the porcine circovirus-like viruses. Virus strains referred to by codename and year of isolation are listed in Table 1 as follows: 01 ~ 06: P1 virus; 07: P2 virus; 08: P4 virus; 09: PCV1; 10 ~ 13: PCV2a; 14 ~ 17: PCV2b; 18 ~ 21: PCV2c; 22 ~ 25: PCV2d; 26 ~ 29: PCV2e; 30 ~ 33: PCV2f; 34 ~ 37: PCV2g; 38 ~ 41: PCV2h; 42 ~ 45: PCV2i; 50: PCV4. The timescale is measured in years.

The tMRCA estimated using the Bayesian SkyGrid model or the Bayesian Skyline model based on the length of the P1 nucleotide sequences may vary slightly. Based on the Bayesian SkyGrid model, the tMRCA estimates are as follows: all P1 strains with 114 or 122 aa Cap in Group A diverged first (0.1702–0.6243; 95%HPD = 0.2896 to 3.3729), followed by the P2 strain (1.9588; 95%HPD = 0.6307 to 6.4755), PCV2a strains (2.5412; 95%HPD = 0.918 to 8.9149); P1 strain with 163 aa Cap and PCV2i strain (5.126; 95%HPD = 1.8426 to 16.1809); PCV2f strains (7.274; 95%HPD = 3.2672 to 22.9108); and PCV2h strains (7.900; 95%HPD = 3.3877 to 30.3241). The tMRCA between Group A and Group B (including PCV2d strains, PCV2g strains, PCV2b strains, and P4 strain) was 9.3693 (95%HPD = 4.1261 to 32.3077). The tMRCA for Group C was 15.1829 to 22.9525 (Figure 3).

The divergence times obtained using the fossil-calibrated MEGA molecular clock and the substitution-rate-based BEAST molecular clock do not yield identical results, such as the relative origination times of PCV2b and PCV2d. Overall, PCVL258 and PCVL264 appeared earliest, followed by the P1 strain, P2 strain, and some genotypes of PCV2, with PCV2c and PCV2e appearing the latest.

### 3.3 Ancestral sequence reconstruction

Based on the phylogenetic tree, the Ancestors tool in MEGA 7 was utilized to reconstruct the most probable ancestral sequences of PCVs using the Maximum Likelihood method with the Tamura-Nei model. Regions containing gaps and missing data were excluded. For each branch, nucleotides with over 50% coverage among the species were considered reliable. We also utilized available web servers to predict the structures of the most probable ancestral sequences.

For all 50 PCVs, we performed ancestral sequence reconstruction (ASR) to determine their ancestral states. The most probable ancestral sequence of PCVs, determined using the Maximum Likelihood method with the Tamura-Nei model, consists of 182 nucleotides, as detailed below.

GGTAAGTGGG GGGTCTTTAA GATTAAATTC TCTGAATTGT ACATACATGG TTACACGGAT ATTGTAGTCC TGGTCGTATA TACTGTTTTC GAACGCAGTG CCGAGGCCTA CGTGGTCTAC ATTTCTAGAG GTTTGTATCC TCAGCCAAAG CTGATTCCTT TTGTTATTTG GTTGAAGTAA TC.

**Figure 4:**
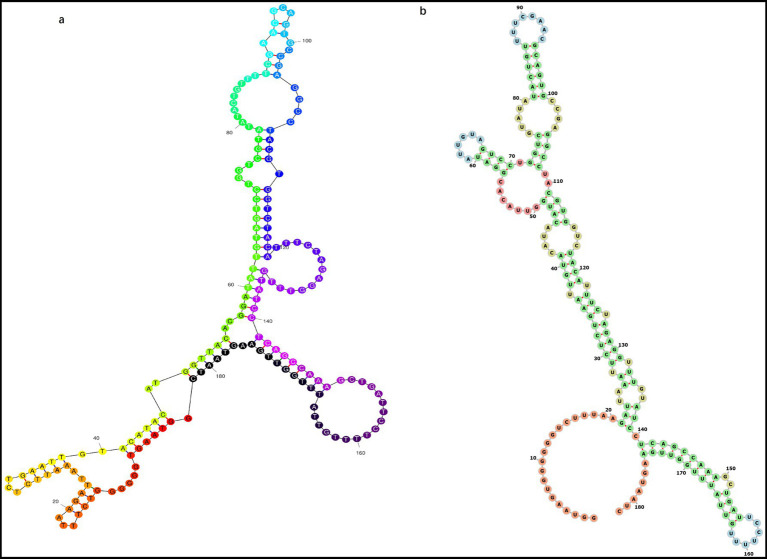
Most probable secondary structure of the original ancestral genome of PCVs containing 182 nucleotides predicted using different online tools. Structures predicted include **(a)** Y-shaped and **(b)** rod-shaped.

Given that PCVs possess circular genomes and linear genomes are prone to degradation, we infer that the PCV ancestral sequence also exhibits a circular genome. We compared the most likely structural models of ancestral sequence predicted by website 1 and found that they exhibit similar irregular Y-shaped folding structures. In contrast, the secondary structure predicted by website 2 and website 3 are consistent, with minimum free energy of −38.90 and −42.88 kcal/mol, respectively. In contrast to the secondary structure predicted by website 1, which displays an irregular Y-like shape, the secondary structure predicted by website 2 and 3 features a rod-like configuration (Figure 4).

### 3.4 Protein structure analysis

Amino acid sequences of the viral Cap proteins from 46 PCV strains were aligned. Compared to early-evolved strains, such as P1, P2, PCV2a, and PCV2i, mutations were observed at residues D77N/G, K88P, I89L/R, and I91V/L in P4, other genotypes of PCV2, and PCV1. The T120S and Q136L mutations were detected in P4, all genotypes of PCV2, PCV1, and P1 with a 163-aa Cap, compared to those in other P1 strains and P2. These results indicate that the compositions of B cell epitopes in early- and late-evolved viral strains have changed.

To analyze whether mutations in the PCVs Cap protein, particularly at sites 77, 120, and 136, affect their structure, we conducted a comparative analysis of the three-dimensional structures of PCVs capsid proteins. The predicted protein conformations for the four circoviruses here exhibit high confidence (Table 2).

**Table 2:** The confidence score of the top-ranked model for different viral capsid proteins in I-TASSER simulations.

Viral cap	GenBank no.	C-score	Estimated TM-score	Estimated RMSD
P1	EF514716	0.56	0.64 ± 0.13	5.3 ± 3.4 Å
P1	OL581721	1.38	0.91 ± 0.06	2.3 ± 1.8 Å
P2	EF514717	0.77	0.62 ± 0.14	7.3 ± 4.2 Å
PCV2d	OR533478	0.68	0.63 ± 0.14	7.1 ± 4.2 Å

The three-dimensional structure of P1Cap with 114 aa was relatively simple, consisting of only one *α* helix (6IDDF9) and two β sheets (31VKVEF35; 92HPNLF96), and differed significantly from that of the later-evolved circoviruses.

The domain of P1 Cap with 163 aa contained one α helix (7DDF9) and eight β sheet structures (1MMR3; 24EYYRI28; 31VKVEFWP37; 48VGSSAV53; 78HTIP81; 87HSRYF91; 128LGTAF132; 141YNIRVTMYVQFR 152).

The P2 Cap contained two α helices (76IDDF79; 85GTN87) and nine β sheets (45FNTRLSRTFGYTV57; 67WAVDMMR73; 94EYYRI98; 100KVKVEFWP107; 118VGTTAV123; 148HTIP151; 157HSRYF161; 198LGTAFE203; 209QDYNIRVTMYVQFR 222).

The PCV2d Cap domain contained one α helix (76INDF79) and nine β sheets (45FNTRLSRTIGYTV57; 67WNVDMM72; 94EYYRI98; 100KVKVEFWP107; 118VGSTAV123; 147RHTIT151; 157HSRYF161; 198LGTAFE203; 209QDYNIRVTMYVQFR 222).

**Figure 5:**
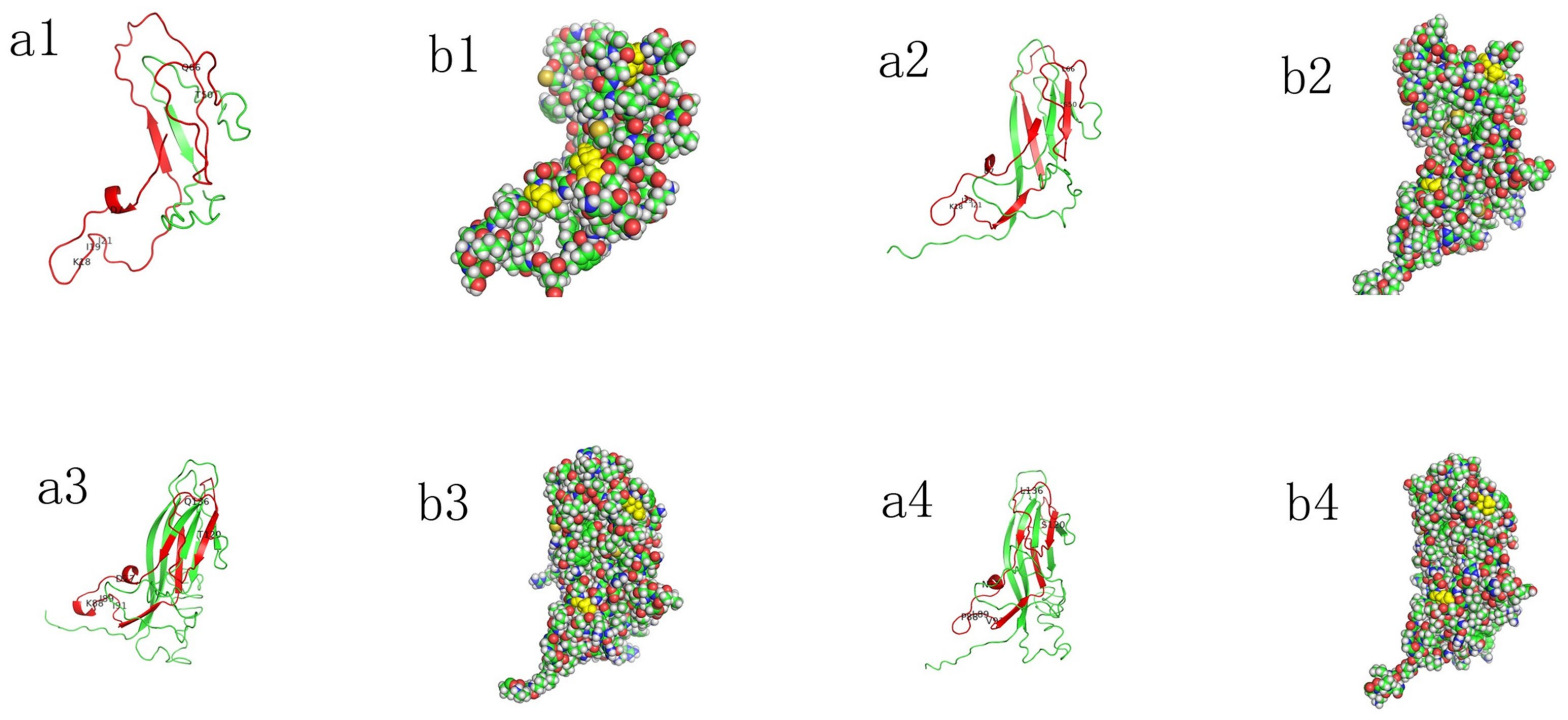
Different evolutionary phases mapped on the 3D structures of PCVs Cap proteins, including P1 (GenBank No. EF514716; OL581721) (1,2), P2 (GenBank no. EF514717) (3), and PCV2d (GenBank no. OR533478) (4). The 3D structure of P1 and P2 Cap was generated via homology modeling using the PCV2 Cap as a template (PDB ID: 3R0R). Structures were visualized with Pymol version 3.0.3. The red region highlights the part of the capsid protein with high amino acid homology, and mutated amino acid sites are labeled **(a)**. Selected residues 77, 120, and 136 are represented as spheres and colored in yellow **(b)**.

Structural analysis showed that amino acid sites 77, 120, and 136 were exposed on the outer surface of P1 Cap with 114aa, whereas site 120 was concealed within P1 Cap with 163 aa, P2 Cap, and PCV2d Cap (Figure 5). This indicates that amino acid sites 77 (D to N or G) and 136 (Q to L) in early-evolved strains did not affect the structure of the late-evolved strains, whereas amino acid site 120 (T to S) may play a significant role in the evolution of PCVs.

## 4 Discussion

Although phylogenetic analysis is a powerful tool, a wide range of inference methods exist. We analyzed selected circovirus sequences, including complete and trimmed genome sequences, using both MEGA and BEAST to investigate the molecular genetic relationships and the origin of PCV2. MEGA was utilized for initial sequence alignment and to perform preliminary phylogenetic analyses, and BEAST was then employed to generate the MCC tree, incorporating molecular clock to provide a more robust estimation of divergence times.

Regardless of the method employed (ML, NJ, and ME) or whether sequences were trimmed, the phylogenetic trees consistently showed similar results. The results indicated that PCV2a is closely related to PCVL258, PCVL264, P1 strains with 114 or 122 aa Cap, and P2; PCV2i is closely related to P1 strain with 163 aa Cap; and PCV2d is closely related to PCVL201, PCVL347, and P4. In contrast, PCV2c and PCV2e are distantly related to other PCV2 genotypes.

Chronological information from previous studies, including timetree, the evolutionary timescale database, provides crucial insights for understanding the evolutionary history of these lineages. Internal nodes represent the most recent common ancestors (MRCA) of at least three evolutionary lineages in a phylogenetic tree. However, it should be noted that estimating divergence times from molecular data is a complex task.

We used the fossil records dated to 1985 for PCV2a, 1999 for PCV2d, 1980 for PCV2c, and 2014 for PCV2e to estimate the divergence times of PCVs through MEGA software. Although relying solely on two fossil records which may not be the oldest could lead to underestimation. Meanwhile, we estimated the PCVs divergence times using substitution rates through BEAST software. Given that the first detection time of the viral sequences may not represent their true existence time, we employed a substitution rate calibration instead of fossil records or tip-dating calibration. Furthermore, considering the variation in substitution rates among different circoviruses, we adopted a relaxed molecular clock model rather than a strict molecular clock model. The overall trend in viral divergence times remains apparent and convincing. The results indicated that PCVL258 and PCVL264, which lack protein-coding capability, evolved first and are considered the common ancestor of PCVs. Subsequently, the P1 virus and P2 virus appeared, followed by PCV2a and PCV2i, while other PCV2 genotypes evolving later.

Furthermore, based on the ancestral sequence reconstruction, we hypothesize that PCVs originated from a nucleic acid agent containing 182 nucleotides, which cannot encode proteins. This agent may possess a circular genome with a secondary structure resembling either a Y-shaped or rod-like configuration resembling a viroid structure (35).

The Cap protein is the sole structural protein. Previous studies have revealed that PCV2 contains at least seven B cell epitopes, which include residues 69–83, 117–131, 122–136, 156–162, 175–192, 195–202, and 231–233 (36–38). Since the three-dimensional (3D) structures of P1 and P2 viral Caps have not been resolved, we modeled the 3D structures of these proteins using homology modeling based on the PCV2 Cap structure (PDB ID: 3R0R) via an online service. Notably, two- point mutations (D77N/G and Q136L) in the B cell epitope region of the PCV2 Cap do not affect the viral structure, whereas the T120S mutation may play a significant role in the evolutionary history of PCVs. Reports indicate that mutations in two amino acids of the PCV2 capsid protein lead to enhanced viral replication *in vitro* while attenuating viral virulence *in vivo*. Additionally, reports suggest that the C-terminus of the PCV2 capsid protein (^227^K) plays a critical role in viral entry into host cells, proliferation, and assembly. Mutations in the amino acids of the PCV2 capsid protein can generate novel neutralizing epitopes, while the residues 106WPCSPITQGDRGVGSTAV123 are identified as a decoy epitope (39–42). The impact of the amino acid change at position 120 in late-evolved PCV strains on viral cell invasion, virulence, and epitope properties requires in-depth investigation.

Studying the origin of life inevitably raises the classic question of which came first: proteins or nucleic acids? While there are two “extreme organisms” in nature-viroids that do not encode proteins and prions that lack nucleic acids, the evolutionary history of these organisms does not resolve the question of whether proteins or nucleic acids came first. The current RNA world hypothesis may address this question, as RNA not only stores information like DNA but also catalyzes reactions similar to proteins (43).

The emergence of porcine circovirus-like viruses and porcine circovirus-like mini agents offers a nearly complete evidence chain for studying the evolution of PCVs. Specifically, PCV2, porcine circovirus-like viruses, and porcine circovirus-like mini agents may have originated from a small DNA molecule lacking protein-coding ability that recombined with other biological genomes. It remains unclear whether this ancestral small DNA molecule had ribozyme-like functions. However, analysis of the early-evolved PCVL258, P1, and P2 genomes revealed that their recombinant nucleotide sequences contain palindrome structures and identical 16-nucleotide fragments (CGTTACTAGTGGATCC), suggesting that recombination was not random and may have involved enzymatic activity. Certainly, the presence of the putative 182-nucleotide fragment has not yet been detected. Additionally, there is no experimental evidence that this fragment can recombine with other biological entities to form circoviruses. Overall, the evolution of PCV may begin with an ancestral sequence, followed first by the emergence of porcine circovirus-like mini-agents, then porcine circovirus-like viruses, and finally different genotypes of PCV2.

## 5 Conclusion

In summary, this study hypothesizes that PCV2 may have originated from small non-coding DNA molecules. This also offers new perspectives on the longstanding question of whether nucleic acids (in DNA form) or proteins emerged first in evolution.

## Data Availability

The datasets generated during and/or analyzed during the current study are available from the corresponding author on reasonable request.

## Footnotes

1
https://github.com/beast-dev/tracer/releases/latest

2
https://zhanggroup.org/I-TASSER
