# Supplementary material for: Phylogenetic analysis of porcine circovirus 2 and its related viruses
Source: Front Vet Sci. 2025 Dec 8;12:1711361. doi: 10.3389/fvets.2025.1711361 (PMC12723004; doi:10.3389/fvets.2025.1711361)
Supplement: Supplementary file 1 [file Image_1.pdf]

Figure legends

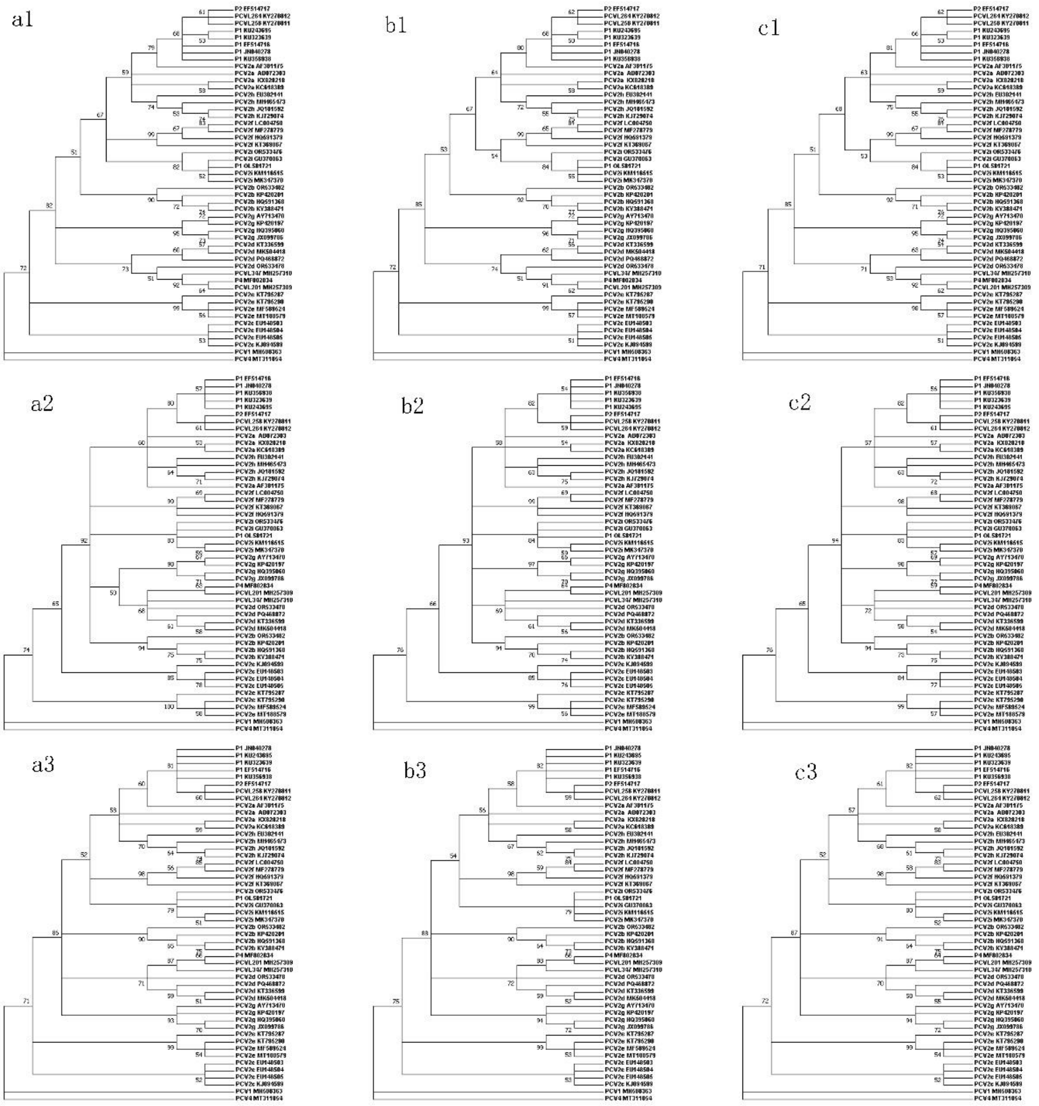

SUPPL FIG1

Phylogenetic tree of the 50 PCV strains in our study based on trimmed sequences based on P1 virus genome length (1); complete genomic sequence (2); and trimmed sequences based on PCVL347 genome length (3). Phylogenetic trees were constructed using Maximum Likelihood (a); Neighbor-Joining (b); and Minimum-Evolution (c) methods in MEGA 7.0, applying the Tamura-Nei; p-distance; and p-distance models. The reliability of the trees was assessed by 1000 bootstrap replications. The values along the branches represent bootstrap values.

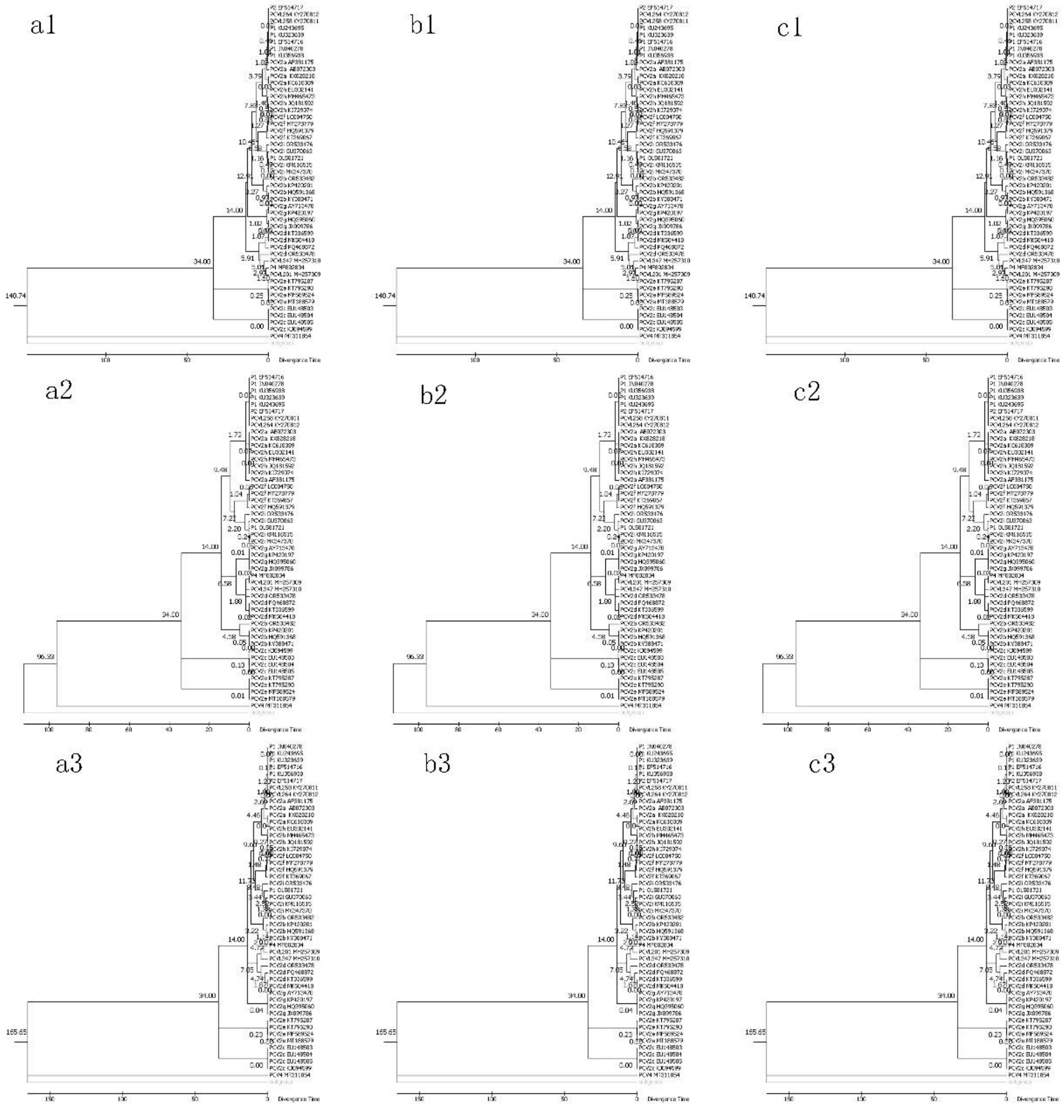

SUPPL FIG2

Evolutionary relationship of taxa (timetree) inferred using the Reltime method based on trimmed sequences based on P1 virus genome length (1); complete genomic sequence (2); and trimmed sequences based on PCVL347 genome length (3). Estimates of branch lengths were derived using ML (a), NJ (b), and ME (c) methods. The timetree was computed using two calibration constrains, and the analysis involved 50 PCV nucleotide sequences. Evolutionary analyses were conducted in MEGA 7.0.
